# Atomic Force Microscopy of Photosystem II and Its Unit Cell Clustering Quantitatively Delineate the Mesoscale Variability in *Arabidopsis* Thylakoids

**DOI:** 10.1371/journal.pone.0101470

**Published:** 2014-07-09

**Authors:** Bibiana Onoa, Anna R. Schneider, Matthew D. Brooks, Patricia Grob, Eva Nogales, Phillip L. Geissler, Krishna K. Niyogi, Carlos Bustamante

**Affiliations:** 1 California Institute for Quantitative Biosciences, University of California, Berkeley, California, United States of America; 2 Biophysics Graduate Group, University of California, Berkeley, California, United States of America; 3 Department of Plant and Microbial Biology, University of California, Berkeley, California, United States of America; 4 Howard Hughes Medical Institute, University of California, Berkeley, California, United States of America; 5 Physical Biosciences Division, Lawrence Berkeley National Laboratory, Berkeley, California, United States of America; 6 Department of Molecular and Cell Biology, University of California, Berkeley, California, United States of America; 7 Life Sciences Division, Lawrence Berkeley National Laboratory, Berkeley, California, United States of America; 8 Department of Chemistry, University of California, Berkeley, California, United States of America; 9 Department of Physics, University of California, Berkeley, California, United States of America; 10 Kavli Energy NanoSciences Institute at the University of California, Berkeley and the Lawrence Berkeley National Laboratory, Berkeley, California, United States of America; University of Hyderabad, India

## Abstract

Photoautotrophic organisms efficiently regulate absorption of light energy to sustain photochemistry while promoting photoprotection. Photoprotection is achieved in part by triggering a series of dissipative processes termed non-photochemical quenching (NPQ), which depend on the re-organization of photosystem (PS) II supercomplexes in thylakoid membranes. Using atomic force microscopy, we characterized the structural attributes of grana thylakoids from *Arabidopsis thaliana* to correlate differences in PSII organization with the role of SOQ1, a recently discovered thylakoid protein that prevents formation of a slowly reversible NPQ state. We developed a statistical image analysis suite to discriminate disordered from crystalline particles and classify crystalline arrays according to their unit cell properties. Through detailed analysis of the local organization of PSII supercomplexes in ordered and disordered phases, we found evidence that interactions among light-harvesting antenna complexes are weakened in the absence of SOQ1, inducing protein rearrangements that favor larger separations between PSII complexes in the majority (disordered) phase and reshaping the PSII crystallization landscape. The features we observe are distinct from known protein rearrangements associated with NPQ, providing further support for a role of SOQ1 in a novel NPQ pathway. The particle clustering and unit cell methodology developed here is generalizable to multiple types of microscopy and will enable unbiased analysis and comparison of large data sets.

## Introduction

Plants are exposed to fluctuations in light quantity and quality, and therefore need to balance productive photochemistry and dissipative photoprotection. This goal is achieved by dynamic regulation of the structure and organization of pigment-proteins throughout the thylakoid membrane. In particular, photosystem II (PSII) and its closely associated light-harvesting complex II (LHCII) form supercomplexes within the grana that undergo reversible molecular modifications and large-scale rearrangements [1]. In *Arabidopsis* thylakoids, the major type of supercomplex found is identified as C_2_S_2_M_2_, and consists of two reaction center core (C) complexes, two strongly (S) bound LHCII trimers, and two additional moderately (M) bound LHCII trimers; supercomplexes lacking one or both M-trimers are also observed (C_2_S_2_M and C_2_S_2_, respectively) [2]. Energy dissipation mechanisms are collectively measured and referred to as non-photochemical quenching (NPQ) of chlorophyll fluorescence, and different components can be distinguished by their kinetics and dependence on specific factors [3].

Electron and atomic force microscopies have been valuable tools for characterizing the global morphology and the distribution of photosynthetic complexes within the thylakoid membrane. In particular, AFM scanning is gentle enough to preserve the membrane integrity, and the resolution is high enough to comfortably assign PSII supercomplexes and characterize their spatial distribution [4], [5]. Through the use of these techniques, it has become clear that PSII complexes are concentrated in the grana region of the thylakoid and are predominantly randomly organized under optimal photosynthetic conditions [6]. In addition, PSII particles are often observed in 2D crystalline arrays; more than 100 distinct sets of unit cells lattice parameters are described in the PSII crystalline array literature, each comprising a single C_2_S_2_, C_2_S_2_M, or C_2_S_2_M_2_ particle with a different placement and orientation [7]–[14]. Low light conditions have been shown to promote the formation of PSII crystals, and excess light reduces the prevalence of these arrays [11], [12], [15]. The presence of crystalline arrays has also been linked with other environmental conditions, e.g. temperature [5], [16] and the plant's genetic background [8], [10], [14], [15],[17]–[19]. Theoretical calculations and Monte Carlo simulations have yielded a thermodynamic phase diagram of model PSII and LHCII complexes, which predicts a pure-fluid region that covers optimal light conditions and a fluid-crystal coexistence that covers regions in low light conditions [20].

Because many grana membranes appear to be at crystal-fluid phase coexistence in vivo, separating particles with crystalline local environments (''crystalline particles'') from those with disordered local environments (''disordered particles'') is a common step in the analysis of nano-resolution micrographs, even when the crystalline unit cell has not been previously characterized. Although qualitative distinctions have been drawn between sets of C_2_S_2_M_2_ unit cells [12], [21], to our knowledge, no authors have previously presented an objective, quantitative method for separating the unit cells for each particle type into structurally distinct classes. In the language of statistical learning [22], this separation is an unsupervised classification task that can be broken down into three steps: (i) develop a multidimensional feature set to represent key aspects of the data (in this case, the positional relationships between a PSII particle and its nearest neighbors); (ii) cluster the data points into an appropriate number of classes; (iii) classify newly-observed data points as belonging to one or another of the classes identified in step (ii). Following this procedure, we present here an intuitive yet statistically grounded taxonomy of PSII unit cell classes, and tools for classifying PSII particles into one of these classes [23].

To demonstrate the usefulness of our PSII unit cell analysis methodology, we applied it to a high-resolution AFM data set of wild-type (WT) and *suppressor of quenching 1 (soq1)* grana membranes from *Arabidopsis*. SOQ1 is a recently discovered thylakoid protein that functions in a novel NPQ pathway [24]. Plants lacking SOQ1 are capable of maintaining effective light harvesting in optimal light conditions, yet exhibit significantly more quenching under high light stress. The molecular mechanism of SOQ1 function, and any nano- or micron-scale structural signatures of this function, are unknown. Due to the important relationship between the structural arrangement of thylakoid membrane pigment-proteins complexes and energy dissipation, we hypothesized that differences between WT and *soq1* membranes may give us insights into the mechanism of this new quenching pathway.

WT and *soq1* grana membranes collected before and after exposure to excess light were imaged by high-resolution AFM, and it was found that our analysis is robust enough to be consistent with previous reports of total crystallinity in WT, while powerful enough to quantitatively discriminate between coexisting PSII crystals. Based on detailed analysis of nearest-neighbor organizational motifs within the crystalline and disordered phases of PSII complexes, we determined the first PSII nanoscale structural signatures of *soq1* mutant membranes and compared them with those known for WT. Our findings suggest that protein-protein interactions are altered in the absence of SOQ1, leading to changes in typical PSII separations in the fluid phase and in typical PSII unit cells in the crystalline phase. These structural properties are likely to affect the reorganization dynamics of PSII and LHCII during illumination, and thus the photoprotective responses.

## Results

### Membrane characterization

PSII-enriched (grana) membranes were prepared from Arabidopsis leaves of the WT and *soq1* mutant grown in control light (175 µmol photons m^−2^ s^−1^) or exposed to photoinhibitory high light (1,200 µmol photons m^−2^ s^−1^ for 90 min).

**Figure 1:**
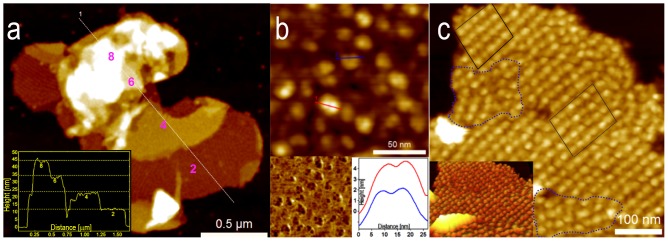
AFM characterization of grana membranes. (**a**) Typical fused grana membranes displaying various levels of thylakoid stacking. White line is a cross-section site with a height profile represented in the inset. As many as eight different membrane layers form four stacked grana discs. Z scale 0–60 nm. (**b**) A high resolution image of a grana disc reveals individual PSII supercomplexes protruding from the membrane. AFM phase contrast image resolves internal structure in each supercomplex (left inset). The cross-section profile on individual complexes (red and blue lines) distinguishes two prominent peaks per complex separated by 9 nm (right inset). (**c**) Grana membranes can be composed of semicrystalline arrays of complexes (marked by black polygons) adjacent to disordered regions (marked by blue dotted lines). Inset shows a 3D representation to facilitate visual array detection. Z scale 0–20 nm.

AFM showed a heterogeneous mixture of grana membrane patches, as shown in Figure 1. Grana isolation conditions were adjusted to enrich the population of intact discs with distinct margins which indicates membrane structural preservation (Figure S1 and Material and Methods). Grana patches varied in shape and size (from 150 nm to 1 µm in diameter). We also found larger, multi-lobed patches consistent with some degree of membrane fusion as reported previously [4]. Most of these patches were double membranes based on their average height of 11 nm, which we refer to as grana discs (Figure 1). Some membrane patches also had higher-order stacking of additional membranes distributed randomly throughout the patch (Figure 1a). The residual upper layers corresponded to partially disrupted grana discs, because their heights are multiples of 11 nm (Figure 1a inset) in agreement with previous reports [4]. These heights were slightly smaller than those obtained when scanning grana membranes in liquid due to dehydration [5]. Our analyses were limited to the double membrane grana discs.

Each grana disc was densely packed with particles, with approximate diameters of 15–25 nm and protrusions above the lipid bilayer of 2–4 nm. High-resolution images revealed internal structure within the particles (Fig 1b, phase inset). For some particles, two prominent peaks were easily detected with a peak-to-peak separation of approximately 7–9 nm (Fig 1b cross-section profile inset). This separation is expected between the two extrinsic oxygen-evolving complexes (OECs) in the dimeric PSII. Thus, we assign these particles as PSII reaction centers protruding from the grana lumenal face in good agreement with other groups [4], [5]. We observed multiple qualitatively distinct classes of PSII organization; Figure 1c shows a representative image of two phases, in which disordered PSII (blue dotted areas in Fig 1c) coexist with 2D crystalline arrays (black boxes in Fig 1c).

### Statistical identification of PSII crystalline unit cells

To determine the local unit cell around each PSII particle, our algorithm fits a Bravais lattice to the coordinates of other particles in the central particle's nearest-neighbor shell (see Materials and Methods). Bravais unit cells are convenient for our analysis because they are intuitive and lower-dimensional than these feature sets, being characterized simply by the lengths *a* and *b* of the two Bravais lattice vectors, and the angle *ϑ* between them. Feature sets for local positional order that are prevalent in the literature, including in the fields of shape-matching [25], [26] and transmission electron microscopy [27], are inherently higher-dimensional. When the coordinates of the neighboring particles are highly ordered, our scheme robustly yields unit cells that agree with unit cells obtained from traditional Fourier transform methods without requiring long-range periodicity. When positional noise is above an upper threshold, the particle is assigned as disordered, thus providing a means of discriminating between ordered and disordered particles. We selected a relatively low threshold, which may lead to an underestimation of the total crystal content of the membranes.

The next key step in our analysis pipeline was developing a taxonomy of unit cell classes using the Gaussian mixture model (GMM) clustering method. A GMM is a probabilistic model that fits several multivariate Gaussian distributions to the data, typically using the expectation-maximization algorithm [28]. GMMs were fit to a training set of unit cells that included crystalline particles picked from our high-resolution (150 nm×150 nm) AFM images, as well as unit cells taken from the literature [7]–[14]. The GMM method alone does not yield information about the number of clusters *k* that produces the most meaningful clustering; additional model selection criteria are necessary if *k* is not known a priori. We used Bayesian Information Criterion (BIC) to select the best value of *k* for many bootstrap-resampled data sets [22]; Figure S2 shows that *k* = 6 was most frequently selected as the best number of clusters. Yet overall, *k* = 6 was selected in only 27% of the bootstrap resamplings, while *k* = 5, 7, and 10 were each selected in 15–20% of resamplings. While this model selection process guided us to present results with *k* = 6 in this work, it also suggests that one should use caution when drawing conclusions about the “ground truth” number and identity of classes of PSII unit cells in this data set. For all three features, the mean *µ* and standard deviation *σ* of each Gaussian mixture component of the non-resampled model fell within the bootstrapped 95% confidence interval (Table S1).

**Figure 2:**
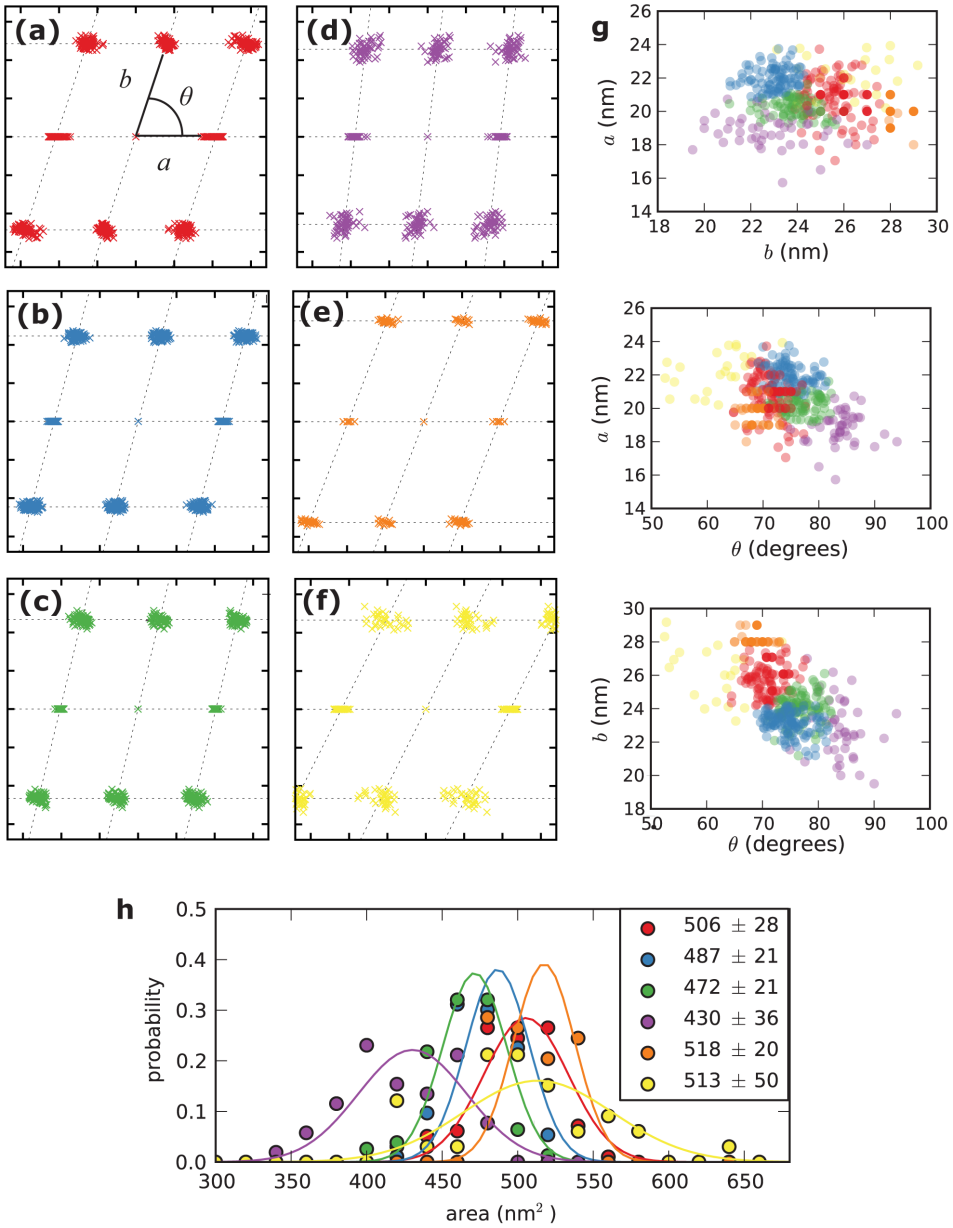
Results of cluster analysis of unit cells in the training data set. (**a**)–(**f**): Fitted Bravais lattices for each particle in the training set, grouped by Gaussian mixture model (GMM) component. The *a* vector of each unit cell lattice is aligned with the horizontal axis. Tick marks are spaced 10 nm apart. Panel (**a**) illustrates the unit cell conventions used throughout this work. **g**: Two-dimensional views of the three-dimensional (*a*, *b*, *ϑ*) data points in the training set, colored by GMM component. **h**: Histograms of unit cell area (area = *ab* sin *ϑ*), grouped by GMM component. Values in legend are means±s.d. in square nanometers; lines are normal distributions with these means and standard deviations.

Figure 2 illustrates the six classes of unit cells that arose from cluster analysis of the training data set. Classes (a) through (f) were ordered by decreasing mixture weight, such that class (a) had the most members (98 particles) and class (f) had the fewest (33 particles). Class (a) is prevalent in the C_2_S_2_M_2_ data set of Kouřil *et al*. (2013), allowing us to assign it as being composed of C_2_S_2_M_2_ particles. Classes (b) and (c) had slightly smaller average areas than class (a); although both are large enough to accommodate a C_2_S_2_M_2_ supercomplex, we cannot rule out the possibility that these classes are composed of slightly smaller supercomplexes (e.g., C_2_S_2_M). We assigned class (d) as C_2_S_2_ because it contained the C_2_S_2_ unit cells [7], [9], [13], had the smallest average area, and was reminiscent of other rectangular C_2_S_2_ unit cells [17], [18]. Class (e) was distinguished by a larger *b* value, which manifests as a larger spacing between rows and the largest average area, and was almost entirely composed of C_2_S_2_M_2_ unit cells like those in Kouřil *et al*.’s “normal light” and “low light” conditions [9]. Class (f) contained the C_2_S_2_M_2_ unit cells from Ref. 9, as well as some outliers with unusually acute angles (*θ*<65°) that may be artifacts of the unit cell determination algorithm.

### Crystal abundances depend on light acclimation and genotype

**Figure 3:**
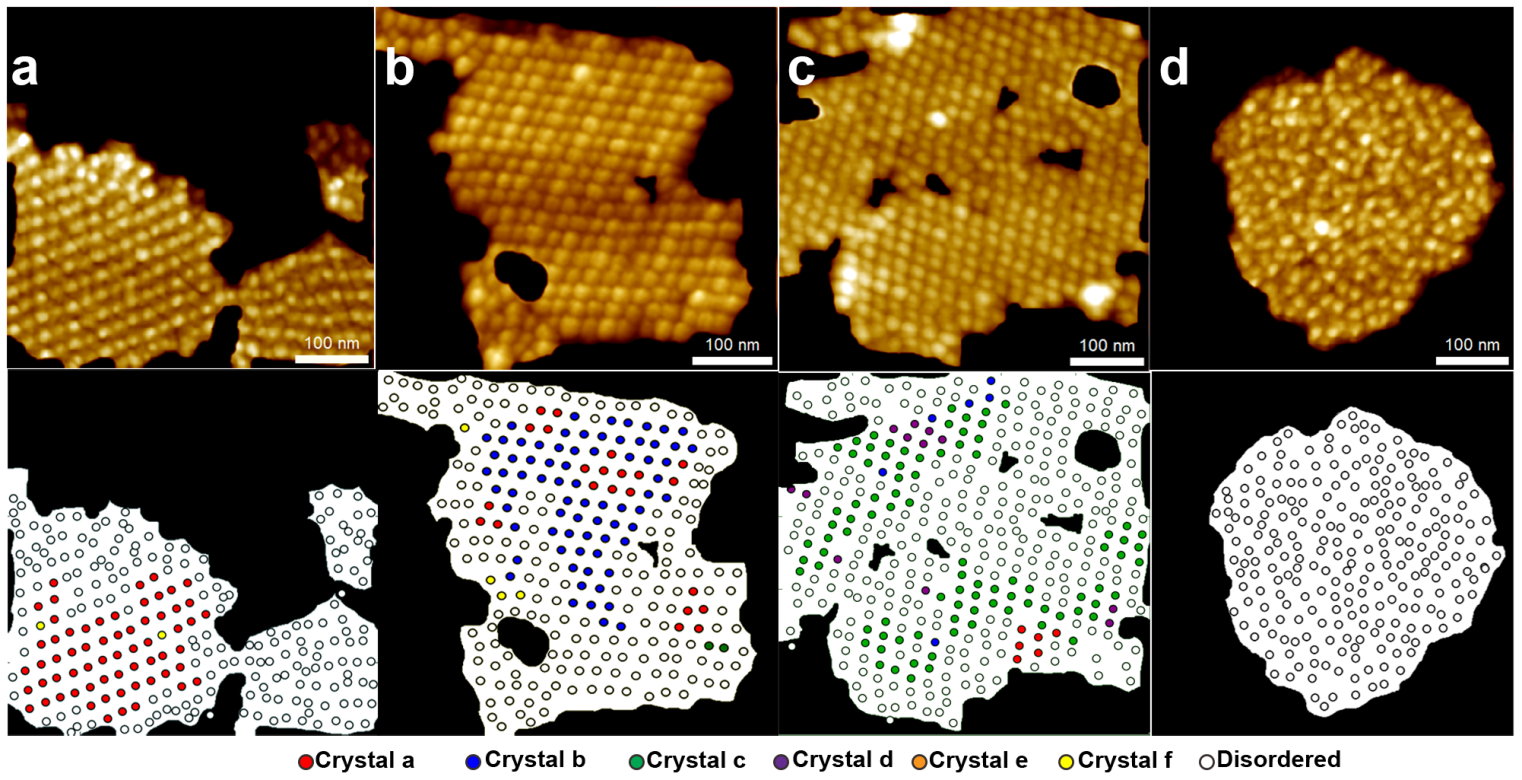
Classification of crystalline particles. Representative masked AFM micrographs (top) and their respective reconstructed images showing results of particle classification (bottom) for (a) membrane enriched with crystal type a, (b) membrane enriched with crystal b (c), membrane enriched with crystal c, and (d) crystal-free grana disc.

To evaluate the effect of inhibitory light on the WT and *soq1* mutant membranes, we collected a larger data set of lower-resolution AFM micrographs (500 nm×500 nm). Each PSII particle in these images was assigned to its maximum-likelihood crystal class based on our GMM, or assigned as disordered if its local unit cell was absent or an outlier. Figure 3 displays some representative AFM micrographs and their equivalent reconstructions with each complex classified. These results clearly demonstrate that our methodology can successfully be applied to lower resolution data.

Examination of the classification results for representative images (Figure 3) revealed a mixture of contiguous disordered regions, contiguous regions of a single crystal type, and regions with small pockets of several crystal types. By eye, the classification results had a very low false positive rate (i.e., few particles that appear to have disordered local environments were assigned to any crystal class) but a higher false negative rate (i.e., the algorithm did not give a crystal label to all particles that a human might assign as crystalline).

**Figure 4:**
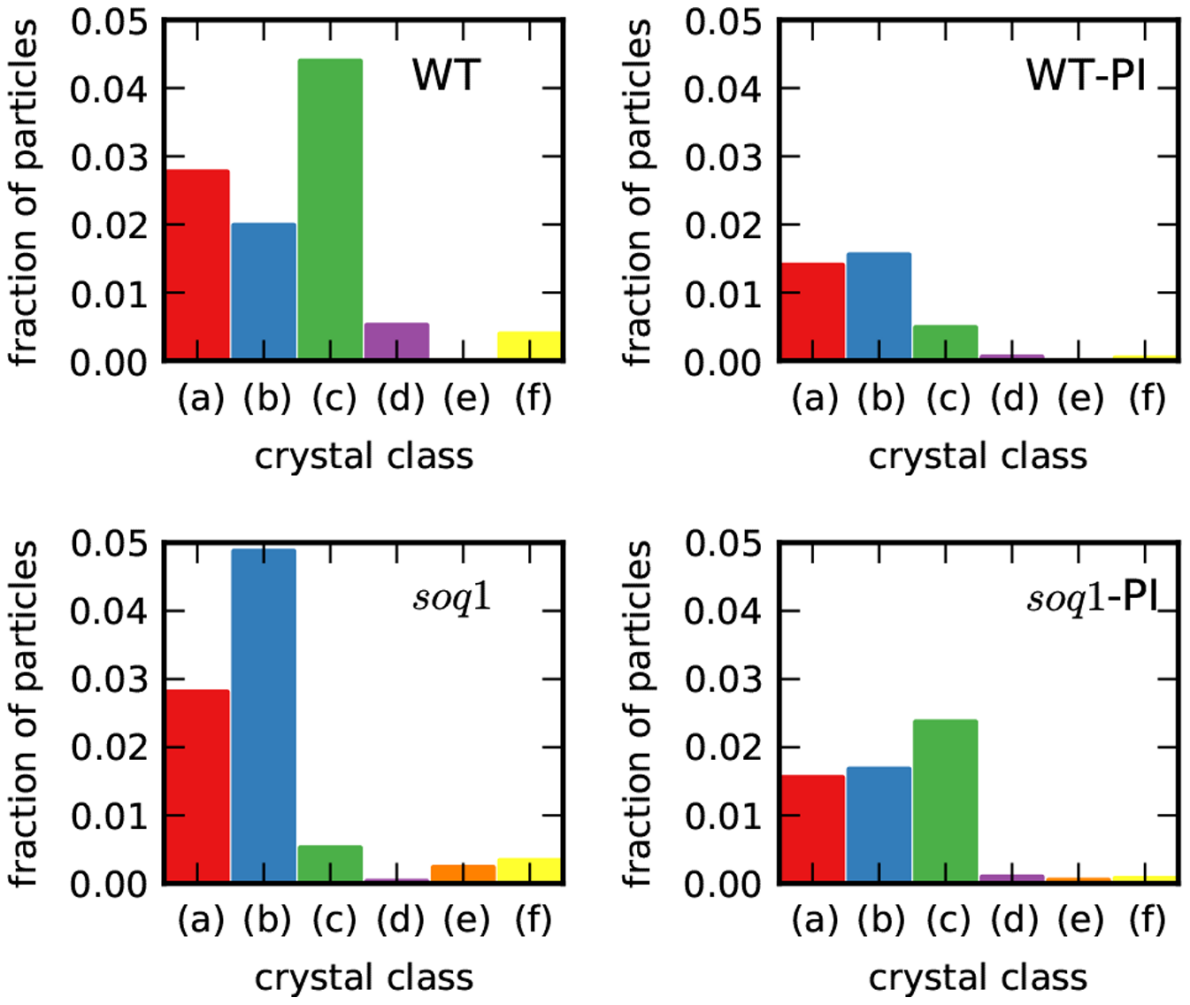
Crystal type distributions in wild-type and *soq*1 grana membranes. Distribution of crystalline particles between crystal classes. Color scheme and class names are as in Figures 2 and 3. The *y* axis indicates the fraction of the total number of particles analyzed; the remaining fraction of particles is disordered. WT = wild-type control light; WT-PI = high-light-treated wild-type; *soq1* = *soq*1 mutant control light; and *soq1*-PI = high-light-treated *soq1* membranes.

**Figure 5:**
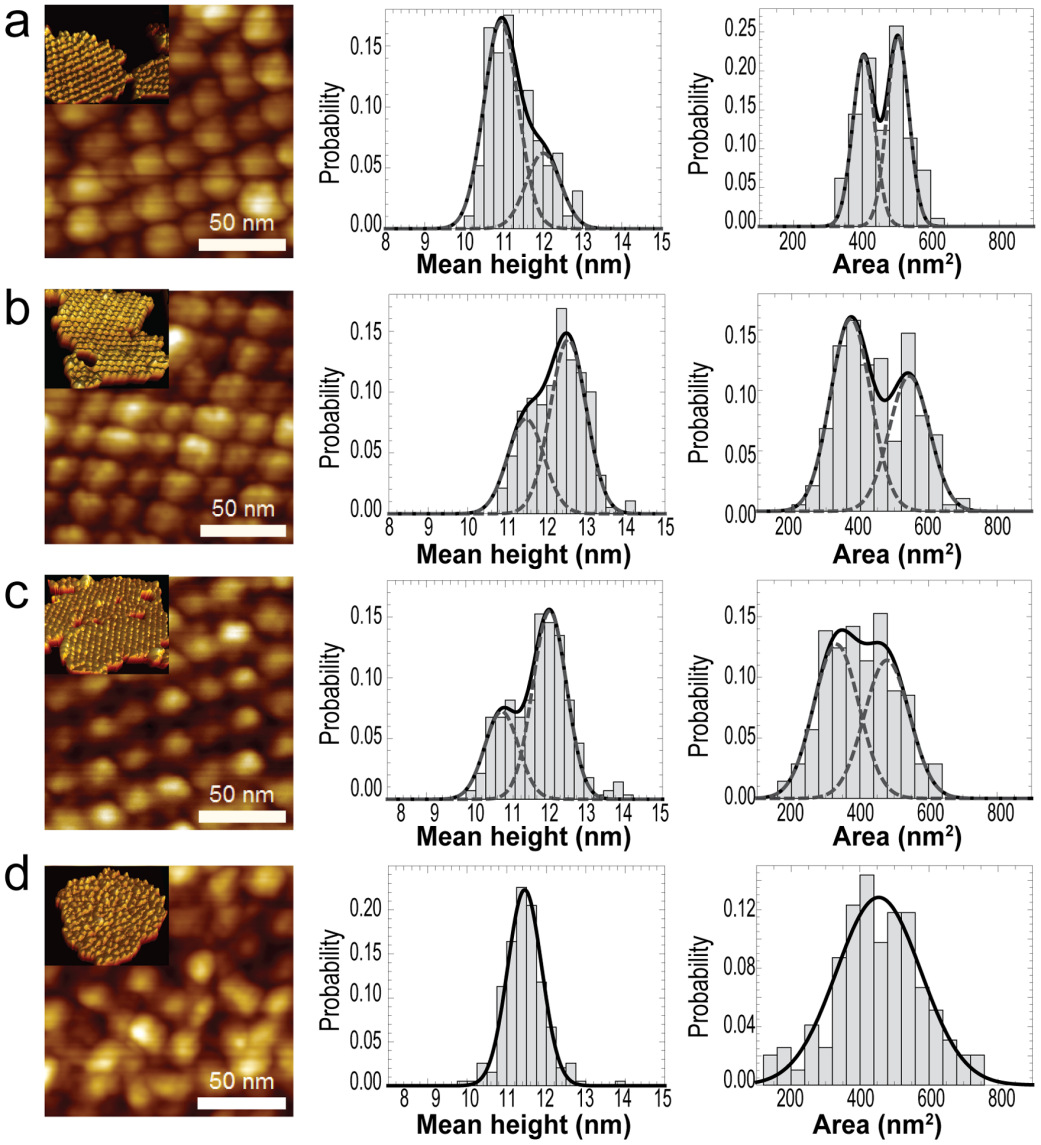
Comparison of particle heights and areas. (**a**)–(**c**) Representative membranes enriched with different type of crystals (a), (b), or (c). (**d**) Crystal-free grana disc. Insets are 3D representations of the entire patch used for the segmentation to obtain the complexes' dimension distributions. *z* scale for all high resolution images 0–8 nm.

**Figure 6:**
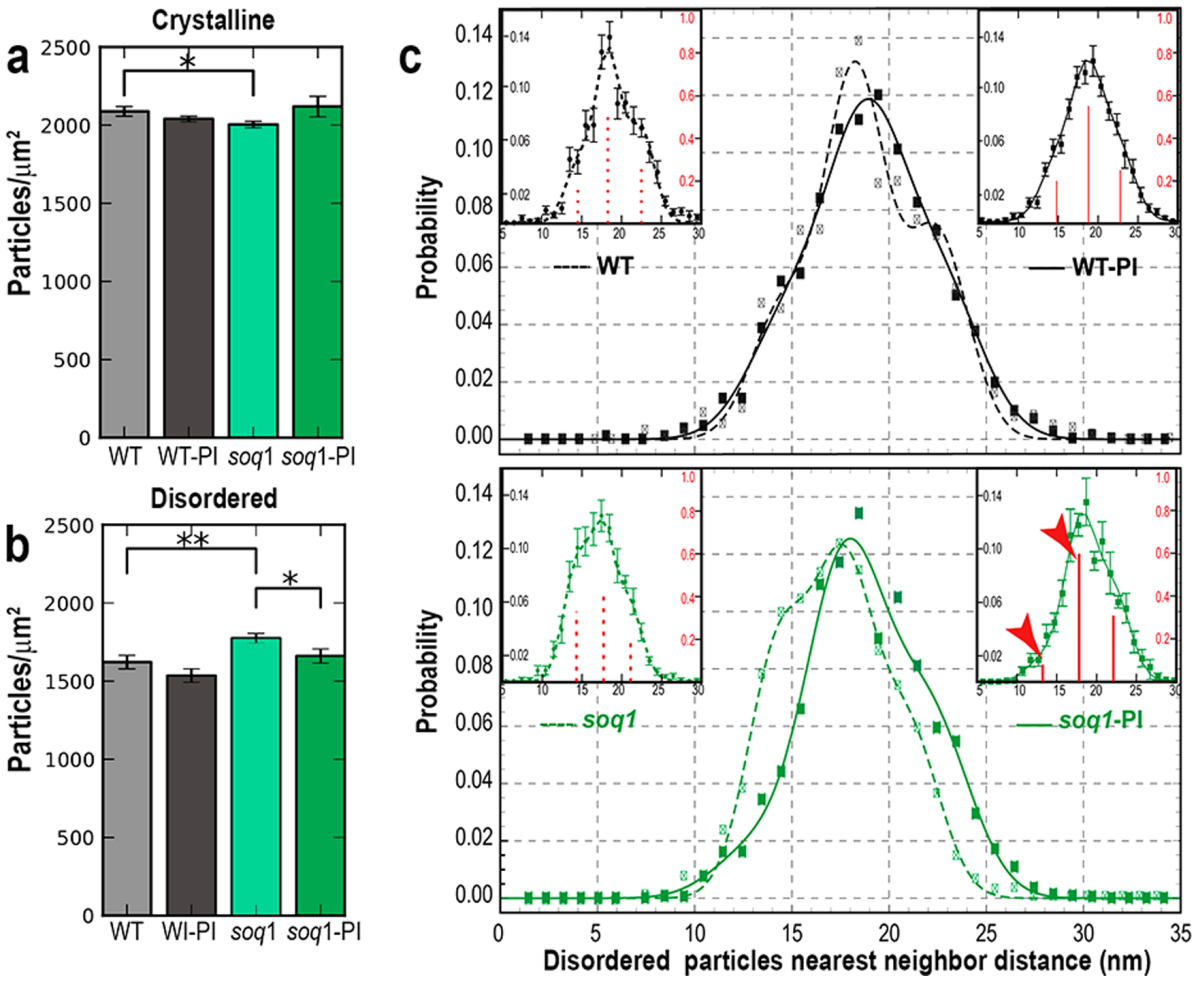
Particle number density and spatial correlations. Particle density in (a) crystalline, and (b) disordered regions. Bars and error bars are means and SEM, respectively, from each experimental condition (N = 5–28 micrographs). All *p*-values are from Welch's t-test; * indicates *p*<0.05, and ** *p*<0.01. (c) Nearest-neighbor distance distributions of disordered complexes in WT, WT-PI (black lines), *soq1*, and *soq1*-PI (green lines). Symbols represent mean nearest-neighbor distance from experimental data. Lines are best multi-Gaussian fits to the data; WT (dashed black), WT-PI (solid black), *soq*1 (dashed green), and *soq1*-PI (solid green). Insets display individual NNDF means and SEM error bars with Gaussian centers and weights indicated by the red spikes (right axes); red arrowheads indicate significant changes in distance distribution upon illumination.

We used the crystal assignments for each AFM image to compare membrane organization between experimental conditions based on several metrics: overall crystallinity and relative occurrence of each crystal (Figure 4), particle size (Figure 5), particle density and spatial correlations in the disordered phase (Figure 6). To measure the net crystallinity in each AFM image, we divided the number of particles assigned to any crystal class by the total number of particles in the image. Under control illumination conditions, we found that WT membranes have 9.2% of crystalline areas, in good agreement with previous reports [12], [29]. The absence of SOQ1 did not strongly modify the crystalline fraction (8.1%). However, crystalline arrays appeared to be slightly more sensitive to high light illumination in WT membranes than in *soq1* membranes: the average net crystallinity decreased from 9.2% to 3.5% in WT membranes, but only from 8.1% to 5.3% in *soq1* membranes (Figure S3). It is worth to noting, however, that the full probability distributions of net crystallinity are broad and not well summarized by their means: at least one membrane patch with >25% crystallinity was observed in every experimental condition, while the majority of images have <5% crystallinity (Figure S3).

The frequencies of different crystal types were also found to depend on the light treatment and the *soq1* mutation. Figure 4 shows the distribution of particles between crystal classes; the remaining particles were disordered. As expected from the clustering model, classes (a), (b), and (c) together accounted for the majority of crystalline particles in each experimental condition, with few particles assigned to classes (d), (e), and (f). The fraction of particles in class (a) was independent of the *soq1* mutation, however photoinhibitory treatment decreased the fraction of (a) by approximately 2-fold. The fraction of particles in class (b) was 0.017–0.020 in all conditions except low-light-acclimated *soq1*, where the fraction was more than 2.5 fold higher. Photoinhibitory treatment had opposite effects on the fraction of particles in class (c) in WT and *soq1* membranes: the fraction decreased in the former and increased in the latter.

Surprisingly, some crystalline domains appeared to alternate rows of wide and tall particles with short and narrow ones (Fig. 3). To investigate this apparent heterogeneity, we complemented our positional analysis by using watershed segmentation on representative patches to compare their particles' size and shape. Indeed, there were significant differences in height and area among particles within the same thylakoid patch (Fig. 5, Table S2). The data were well fitted to normal distributions, and the goodness of fit was done using the Akaike information criterion (AIC). Crystalline regions displayed bimodal distributions in both particle height and area, indicating the presence of at least two distinct types of complexes (Fig. 5a–c). In contrast, particle heights and areas in the crystal-free membranes were each best fit by a single Gaussian (Fig. 5d).

Visual inspection of the AFM micrographs suggested differences in particle densities between experimental conditions. Therefore, we next determined the particle number density in the grana disc regions of each micrograph. Net grana particle densities include contributions from both disordered and crystalline particles, weighted by the relative area occupied by each structural motif. To deconvolute these factors, we used our crystalline classifications to separately calculate the particle number density in the disordered and crystalline membrane regions. WT and mutant membranes displayed statistically different particle densities, as shown in Figure 6ab. In disordered regions, the density difference between WT and *soq1* membranes was very pronounced (Fig. 6b). Another difference was found between *soq1* membranes before and after photoinhibitory light treatment: the particle density in disordered regions decreased upon light treatment. In general, the crystalline particle densities were higher than the disordered densities, and were tightly clustered around 2000–2100 particles/µm^2^ for all conditions (Fig. 6a). Note that 2050 particles/µm^2^ corresponds to 488 nm^2^/particle, approximately the size of a C_2_S_2_M_2_ unit cell (Figure 2h), as expected.

While disordered particle configurations lack the periodic structure of crystalline configurations, they can still feature positional correlations. To investigate the internal organization of the disordered particle configurations, we calculated the nearest-neighbor distribution function (NNDF) for particles in micrographs with no crystalline particles (Fig. 6c). The NNDF for WT was consistent with previously published distributions from *Arabidopsis* and spinach plants grown under similar conditions [4], [12], [29]–[31], confirming that our grana isolation method does not significantly modify the interactions between complexes. NNDFs from all conditions could be fit by a dominant intermediate-distance peak (∼18 nm), with flanking peaks centered at shorter (∼14 nm) and longer (∼22 nm) distances, similar to previous reports [4], [30]; see Table S3 for fitting details. The structure of the NNDF was similar for disordered particles in WT, WT-PI, and *soq1*-PI membranes. Interestingly, shorter nearest-neighbor distances were more common in *soq1* than in the other conditions, while longer distances were less common.

## Discussion

### Single particle unit cell method for detecting, classifying, and comparing local crystalline PSII complexes in *Arabidopsis* grana membranes

Although diverse types of PSII supercomplex crystalline arrays have been detected in both intact and partially solubilized thylakoid membranes from several species for decades, our understanding of the biological implications of such crystalline organization is still developing. Several laboratories have used a variety of high-resolution microscopy techniques to correlate the PSII propensity to form ordered arrays with photon starvation [11], [12], [15], and with alterations to thylakoid protein composition and/or identity [8], [10], [14], [15], [17]–[19]. To achieve a unified understanding of the biophysical causes and consequences of PSII array formation, there is an urgent need for robust and generalizable image analysis methodologies that promote straightforward, quantitative comparisons between the degree and type of PSII crystal formation observed by different groups.

With our crystal classification methodology (Picolo, Point-Intensity Classification Of Local Order), we have developed an objective tool for structural comparisons between membranes exposed to diverse environmental conditions and with different genetic backgrounds. We applied this methodology to detect differences in crystalline structural features between WT and *soq1* mutant *Arabidopsis thaliana* grana membranes treated with control or excess light conditions to test the method's robustness and potentially gain further insights on the structural organization of this mutant.

In control light conditions, our results indicate that, regardless of the genetic background, a small fraction of particles are organized into crystalline arrays (8–10% of total particles) (Fig. S3). Under comparable experimental conditions, our result for WT membranes is consistent with EM studies [12], [29]. Specifically, the average crystallinity by area that we observe is similar to the previously reported fraction of micrographs with any crystal. It has been suggested that detergent solubilization of thylakoids can possibly introduce structural artifacts in the membrane organization. Although we cannot entirely rule out this possibility, crystalline domains have also been observed in freeze-fracture EM images of WT *Arabidopsis* chloroplasts, and the reported fraction of crystalline membranes agrees with those from solubilized membranes, including our results [29]. This indicates that the membrane architecture was likely preserved and that the PSII ordered arrays were not introduced during solubilization. Furthermore, our Picolo training algorithms were tested on unbiased pooled unit cells obtained from our high resolution images and those published by others. If a systematic artifact was introduced, one would expect segregation of those “artifactual” data when the classifier was applied. Our unit cells were well spread across the different crystal clusters in the training set (Figure 2g).

We found that even relatively short exposure (90 min) to high light triggers crystal dissolution in both WT and *soq1* membranes. Our results agree with observations reported for fully high-light-acclimated membranes, in which the fraction of crystal-containing micrographs was severely decreased [12]. Crystallinity in WT membranes appears to be more sensitive to light than in mutant membranes; average net crystallinity decreased by almost a factor of 3 in the former, and only decreased by a factor of 1.5 in the latter.

### Molecular models of different PSII crystalline landscapes support their inherent structural versatility

**Figure 7:**
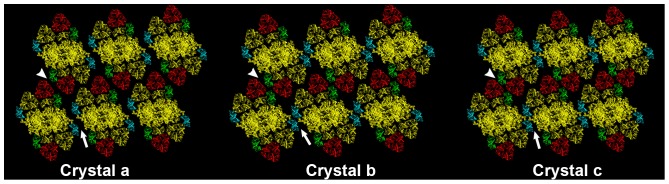
Molecular models of predominant 2D arrays found in *Arabidopsis* wild-type and *soq1* grana membranes. These structures were generated by fitting a molecular model of the PSII C_2_S_2_M_2_ supercomplex [2] into the average unit cell of each class (Table S1), then refined by determining the range of PSII orientations that did not yield steric collision. Reaction center core dimer (C_2_), S-type light harvesting trimers, and minor antenna CP29 are represented in yellow; M-type trimers, CP24, and CP26 antenna monomers are depicted in red, green, and cyan respectively. Inter-supercomplex M-CP24 interactions are indicated by white arrowheads, and CP26 interactions are indicated by white arrows.

Based on the nanoscale AFM data, we propose internal structures for each PSII-LHCII crystal class by placing a C_2_S_2_M_2_ particle at the center of each unit cell and determining particle orientations that do not result in steric clashes. Our models for the dominant classes (a), (b), and (c) are depicted in Figure 7, and the remaining crystal classes are shown in Figure S4. As expected, class (a) was very well fit by the model in Figure 3A of [12], which features end-to-end arrangements of CP26 (cyan) and of CP24 (green) subunits on adjacent supercomplexes, with separations of several nanometers within each pair of minor complexes (white arrows). The best-fit models for class (c) were similar to class (a), but with smaller separations between the peripheral antenna of adjacent supercomplexes. In contrast, the Bravais lattice of class (b) was best fit by a model with face-to-face arrangements of CP26 subunits, and with close end-to-end contacts between CP24 subunits. Rotations of the PSII axis by ±6° were possible within crystal (a) without creating steric clashes, while the smaller unit cells of crystals (b) and (c) led to smaller ranges of rotational freedom (±2° and ±0.5°, respectively). We note that each of these lattices could also be fit by tiling molecular models of smaller supercomplexes, e.g., C_2_S_2_M.

Our crystal classification revealed that photoinhibitory conditions have specific effects on individual types of crystals, and that those effects are different in the absence of SOQ1. The molecular basis by which the recently discovered SOQ1 protein induces high-light NPQs independent of pH gradient, zeaxanthin accumulation, or LHCII phosphorylation are unknown [24]. It is possible that SOQ1 reshapes the PSII crystallization phase diagram, directly or indirectly, in part by stabilizing class (c) relative to class (b) in control light conditions and destabilizing class (c) after prolonged illumination.

The modeled molecular arrays suggest an intriguing hypothesis for the structural modulations that occur in the absence of SOQ1. Crystals (a) and (c) may be able to locally interconvert via slight rotations or translations while maintaining similar antenna contacts. On the other hand, the rotational restriction and distinct minor antenna contacts of crystal (b), which dominates in *soq1* control-light membranes, indicates that it may be stabilized by different protein-protein interactions and that a cooperative transition would be necessary to convert between crystal (b) and the other crystals. Slight changes to the relative organization of chlorophyll transition dipoles can have dramatic effects on fluorescence and energy transfer efficiency and/or pathway [6], [32], [33], so it is conceivable that the distinct CP26 and CP24 contacts in crystal class (b) could have a functional effect.

### Apparent heterogeneity within grana crystals

In addition to particle locations, AFM micrographs contain additional important information about particle size and shape, which we studied by segmentation analysis (Fig. 5). Surprisingly, our AFM data suggests that the identities of the particles comprising lattices of crystal classes (a), (b), or (c) may not be unique. Instead, we found that each lattice can contain a mixture of smaller and larger particles. This variation in particle size within crystals was not detectable based on particle positions alone.

This result is unexpected based on previous EM studies of PSII crystalline arrays, which (to the best of our knowledge) have only observed crystals composed of homogenous PSII supercomplex unit cells. This discrepancy can be explained if the two populations of particles that we detect have differently-sized protrusions above the membrane but indistinguishable projected electron densities, or if multiple populations of electron densities have been present in EM micrographs but tend to be averaged together during data analysis. We cannot rule out membrane distortions during deposition or drying due to surface defects, inhomogeneous electrostatic interactions, or membrane rippling.

Our finding is also intriguing from the perspective of the thermodynamics of crystallization: a perfect crystal contains homogeneous unit cells by definition, and is destabilized by defects that disrupt steric or attractive energetic contacts between particles. On the other hand, inhomogeneities that have a negligible effect on inter-particle interactions will have a negligible effect on crystal formation and stability. For instance, we speculate that mixtures of PSII supercomplexes with identical antenna organization but different extrinsic protein subunits could form co-crystals like those we observe. Such mixtures could occur if variation in extrinsic proteins exists in vivo, as has been suggested [34], or if subunits from some oxygen-evolving complexes in a preexisting crystal were lost during sample preparation. Further EM and/or AFM imaging under different experimental conditions could confirm this observation.

### SOQ1 affects organization of disordered PSII supercomplexes

We find that the *soq1* mutation also affects the local structure within the fluid phase of the grana membrane. In control light conditions, disordered PSII complexes in *soq1* grana are significantly more densely packed than those in WT grana (Fig. 6b). The NNDF of disordered particles in *soq1* membranes reveals that the increase in particle number density is associated with a dramatic increase in the probability of shorter nearest-neighbor distances and a decrease in the probability of longer nearest-neighbor distances (Fig. 6c). These findings point to a role for SOQ1 in affecting PSII density in the grana by disrupting protein-protein interactions that favor smaller PSII separations.

One candidate factor that could be affected by SOQ1 in the absence of photoinhibitory light is attractive interactions between antenna complexes of neighboring PSII complexes. We favor this hypothesis because different contacts between minor antenna complexes are present in the crystals in *soq1* membranes (Fig. 7), because SOQ1 is thought to affect an antenna-associated component of NPQ [24], and because arguments about entropic driving forces are not consistent with a higher PSII density in *soq1* grana. The reducing function of the lumen-localized thioredoxin-like domain of SOQ1 [24] could be involved, directly or indirectly, in a biochemical modification that causes the modulation in antenna interactions.

### High-light stimulation induces PSII reorganization in *soq1* distinct from qE

Upon exposure to photoinhibitory light, PSII density decreased slightly in WT grana and significantly in *soq1* grana (Fig. 6b). This observation is consistent with a net flow of PSII from the grana to the stroma lamellae during the PSII damage-and-repair cycle [15], and agrees with the finding that *soq1* plants are not deficient in PSII repair [24].

Typical separations between disordered PSII complexes are known to depend on changes in light stimulation and on mutations of the qE-associated protein PsbS [29], [30], [35]. We find that the NNDF signature of *soq1* does not match the trends of PsbS deletion or overexpression mutants: locally disordered particles in *soq1* membranes display reduced PSII separations in control conditions, yet have WT-like PSII separations in photoinhibitory conditions. Thus, the changes in local pigment-protein organization within the grana membrane upon enhanced NPQ induction in the absence of SOQ1 appear to be distinct from other described NPQ-correlated reorganizations. Indeed, Brooks *et al.* presented biochemical results indicating *soq1* quenching follows a different, previously undescribed NPQ pathway [24]. In addition, this difference in NNDF suggests that the changes in local organization of disordered PSII upon illumination are far more dramatic in *soq1* than in WT grana.

What is the relationship between the overall PSII organization we characterized and the photoprotective dynamics of *soq1* described previousl [24]. Our findings suggest that *soq1* plants have non-WT-like interactions between PSII complexes under low light. This initial state of *soq1* could predispose the mutant to the formation of a unique quenching pathway upon high-light-induced rearrangement of PSII complexes. Moreover, high PSII densities in *soq1* membranes hamper their rate of diffusion, which could contribute to decelerate the relaxation process when the plants are returned to low light conditions. Further work will be necessary to determine the details of the local structural motifs that are present within the fluid phase of PSII-LHCII complexes in *soq1* grana during NPQ induction and relaxation, the protein-protein interactions that stabilize them, and the kinetics of the transformations between them.

## Conclusions

We present a statistical unit cell analysis methodology to quantitatively characterize protein arrangements. Because the input to our algorithm is the spatial coordinates of a set of particles rather than image data, it can be applied to coordinates extracted from images taken with EM, AFM, or any other imaging modality, or from computer simulations. This generality puts disparate experimental techniques on equal footing, promoting straightforward clustering of and comparison between large data sets.

This method allowed the initial structural characterization of isolated grana membranes from the *soq1* mutant to understand the role of SOQ1 in the thylakoid. Our results indicate that PSII crystalline array formation is not only finely tuned genetically, but that each type of crystal packing is distinctively rearranged upon exposure to photoinhibitory light. SOQ1 appears to play a role in modulating protein-protein interactions among neighboring PSII supercomplexes. In particular, removal of this thylakoid protein appears to favor enhanced attractive PSII interactions reflected by: decreased nearest neighbor distances in the fluid phase, stabilization of smaller lattices in the crystalline phase, and consequently increased particle density on the grana membrane. Certainly, PSII interactions and global organization modifications can have functional implications in photochemical and/or non-photochemical processes.

Our findings open new avenues toward a better understanding of the role of SOQ1 in the thylakoid. Exploring structural characteristics affecting the entire 3D architecture of thylakoids, membrane-lumen interactions, and overall stacked grana morphology will help in dissecting the details underlying this slowly relaxing type of NPQ.

## Materials and Methods

### Sample preparation

Wild-type and *soq1* mutant *Arabidopsis thaliana* plants were grown at 175 µmol photons m^−2^ s^−1^ of light (10 h/day) at 21.5 °C for 8 weeks. The growth of plants and preparation of grana membranes from WT and *soq1* were done at the same time in order to exclude differences that might arise from growth conditions and/or sample preparation. For high-light treated samples, several plants were exposed to 1,200 µmol photons m^−2^ s^−1^ for 90 min before isolating thylakoids. Grana membranes were isolated as described [2] with the following modifications. Leaves from several plants were homogenized in a commercial blender by 8–10 half second pulses and filtered through a 41 µm nylon membrane using a light hand-generated vacuum. Thylakoid membranes were adjusted to a chlorophyll concentration of 2.5 mg/mL and 3/16 volumes of 7.6% (w/v) *n*-dodecyl-α maltoside (α-DM), 15 mM NaCl, 5 mM MgCl_2_ was added and incubated with gentle rocking for 20 min at 4 °C in the dark. The detergent solubilization was carefully adjusted to insure high concentration of isolated but intact grana discs; α-DM for 20 min was used rather than Triton X-100, as this treatment yielded larger and more homogeneous membranes (as shown in Figure S1) in agreement with previous reports [36]. The final sample was frozen immediately in liquid nitrogen as 5 µL aliquots for microscopic inspection.

### AFM data acquisition

Four different types of samples were imaged: WT low-light-acclimated (WT) and photoinhibited (WT-PI) grana membranes; and *soq-1* mutant low-light-acclimated (*soq1*) and photoinhibited (*soq1*-PI) membranes. Grana aliquots were deposited on freshly cleaved mica (10 mM tris-HCl pH 7.5, 150 mM KCl and 25 mM MgCl_2_), and incubated at room temperature for 1–3 hours. Mica was rinsed with water ten times and dried under N_2_ gas flow. AFM measurements were performed with a Multimode AFM Nanoscope V (Bruker Co.). The samples were imaged in tapping mode; the silicon cantilevers (Nanosensors) were excited at their resonance frequency (280–350 kHz) with free amplitudes of 2–15 nm. The image amplitude (set point A_s_) and free amplitude (A_0_) ratio (A_s_/A_0_) was kept at ∼0.8. All samples were imaged at room temperature in air, at a relative humidity of 30%. More than eight different grana patches were scanned per each type of membrane. Bi-layered patches were fully mapped at scans of 500 nm×500 nm. Higher resolution of 150 nm×150 nm images were also recorded.

### Image processing and particle picking

Raw AFM images were flattened and leveled using Gwyddion 2.3 [37]. To determine the height of the membranes and/or particles, bare mica was set at zero nm. Single particle's center of mass were picked from each image. For simplicity, the particle picking was restricted to bi-layer areas by masking out multi-layer and bare mica areas interactively using boxer from EMAN [38]. Initial image processing was done in SPIDER [39]. An average supercomplex profile was obtained by averaging a few hundred supercomplexes extracted from AFM micrographs, followed by rotational averaging. Single particles were located by cross-correlation with the average profile, and peak search. The (x,y) coordinates corresponding to each complexes' center of mass was retrieved. False negatives and incomplete particles were manually removed in boxer. Particle dimensions (mean heights and area) were obtained from particles selected by watershed segmentation (package features) from the particle and pore analysis module included in SPIP. Particle dimension distributions and fittings were done with Wolfram Mathematica. The goodness of fit for normal distributions was done using the Akaike information criterion.

### Feature extraction for local unit cells

For particle *i* at position **r** *_i_*, a local unit cell ⊝*_i_* = (*a_i_*,*b_i_*,*ϑ_i_*) was extracted by the following algorithm.

1. Determine the set *A_i_* of neighboring particles *j* around particle *i*, *A_i_* = [**r** *_j_* | *r*_min_<*r_ij_*<*r*_max_], where the cutoffs *r*_min_ = 14 nm and *r*_max_ = 33 nm are chosen to select the first PSII coordination shell based on typical PSII *g(r)* data. Let *n_i_* be the number of particles in *A_i_*.

2. Find the Bravais lattice *B_i_* centered at **r** *_i_* spanned by the real-space lattice vectors (**u** *_i_*,**v** *_i_*) that minimizes the penalty function *π(i)*

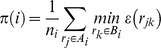
using the piecewise radial error function 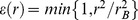 and a cutoff distance *r_B_* = 7 nm. COBYLA [40] was used to minimize *π(i)* subject to the constraints *r*_min_<|**u** *_i_*|<*r*_max_ and *r*_min_<|**v** *_i_*|<*r*_max_.

3. If *n_i_*<4 or *π(i)*>*π*_min_ = 0.2, then ⊝*_i_* does not exist. Otherwise, ⊝*_i_* exists; continue.

4. We can write any point **r** *_k_* in *B_i_* in polar coodinates, **r** *_k_* = *n_uk_* **u** *_i_*+*n_vk_* **v** *_i_* = (*r_k_*,*ϑ_k_*). For any pair of distinct points **r** *_k_*, **r** *_l_* in *B_i_*, define the unit cell ⊝*_kl_* = (*a_kl_*,*b_kl_*,*ϑ_kl_*) by *a_kl_* = *r_k_*, *b_kl_* = *r_l_*, and *ϑ_kl_* = *ϑ_k_*–*ϑ_l_* ∈ [0,2*π*). Let *C_i_* be the set of smallest unit cells, *C_i_* = [⊝*_kl_* | *n_uk_*,*n_vk_*,*n_ul_*,*n_vl_* ∈ [–1,0,1]]. Then we select ⊝*_i_* via
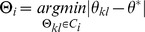
subject to the constraints *a_kl_*<*b_kl_*+*r_e_* and *ϑ*_min_<*ϑ_kl_*<*ϑ*_max_. The angular parameters *ϑ*_min_ = 50°, *ϑ*_max_ = 120°, and *ϑ* ^*^ = 75° were chosen to favor unit cells in the range of the unit cells in [12]; *r_e_* = 1 nm is a small error term on the scale of the pixel size that allows the angular constraints to be satisfied consistently when *a_kl_*≈*b_kl_*. The cutoff parameters were generously chosen to favor a small fraction of false positives (i.e., detection of a unit cell around a locally disordered particle), which could be screened out later during classification.

### Clustering and classification

For clustering, we constructed a training data set consisting of 3 features (*a_i_*, *b_i_*, *ϑ_i_*) for each of 101 unit cells from published PSII crystalline arrays [6]–[10], [12], [14] and 303 unit cells extracted from particles in 31 high-resolution AFM images. The scikit-learn Python package [41] was used to fit diagonal Gaussian mixture models to this data set. To select the number *k* of components in the Gaussian mixture, 1000 bootstrap-resampled data sets were generated and the best value of *k* in the range 1–10 was selected for each data set via BIC (Fig. S2).

For classification, a two-step pipeline was used. First, a probabilistic classifier was constructed from the *k* = 6 Gaussian mixture model using the maximum likelihood decision rule [42]; a rejection cutoff on the class probability of *p_c_* = 0.005 was used to classify outliers into an additional “disordered” class (also occupied by particles for which no unit cell was found). Second, a spatial *k*-nearest neighbor majority-rule filter, where *k* is the number of particles within a radius *r*_max_ of particle *i*, was used on the categorical output of the classifier to reduce the noise.

### Software

We have implemented the algorithms for unit cell identification, clustering, model selection, and classification in Picolo. Picolo is a Python package for analyzing local spatial order in sets of 2d coordinates, which we have made freely available on GitHub [23]. Picolo also includes standard algorithms for rotation-invariant Fourier and Zernike features, support vector machine classifiers, and radial distribution functions.

## Supporting Information

Figure S1 AFM micrographs of grana thylakoid solubilized under different detergent conditions.
(DOCX)

Figure S2 Model selection metrics for the Gaussian mixture model.
(DOCX)

Figure S3 Histograms of net particle crystallinity by AFM image.
(DOCX)

Figure S4 Structural models of the six types of crystal arrays clustered by Picolo analysis package.
(DOCX)

Table S1 Gaussian mixture model parameters, with bootstrapped 95% confidence intervals.
(DOCX)

Table S2 Comparison of grana complexes height and area parameter distributions from representative patches with different type of packing.
(DOCX)

Table S3 Comparison of nearest neighbor distribution's fitting parameters.
(DOCX)
