# Supplementary material for: Atomic Force Microscopy of Photosystem II and Its Unit Cell Clustering Quantitatively Delineate the Mesoscale Variability in Arabidopsis Thylakoids
Source: PLoS One. 2014 Jul 9;9(7):e101470. doi: 10.1371/journal.pone.0101470 (PMC4090009; doi:10.1371/journal.pone.0101470)
Supplement: Figure S1 — AFM micrographs of grana thylakoid solubilized under different detergent conditions. (DOCX) [file pone.0101470.s001.docx]

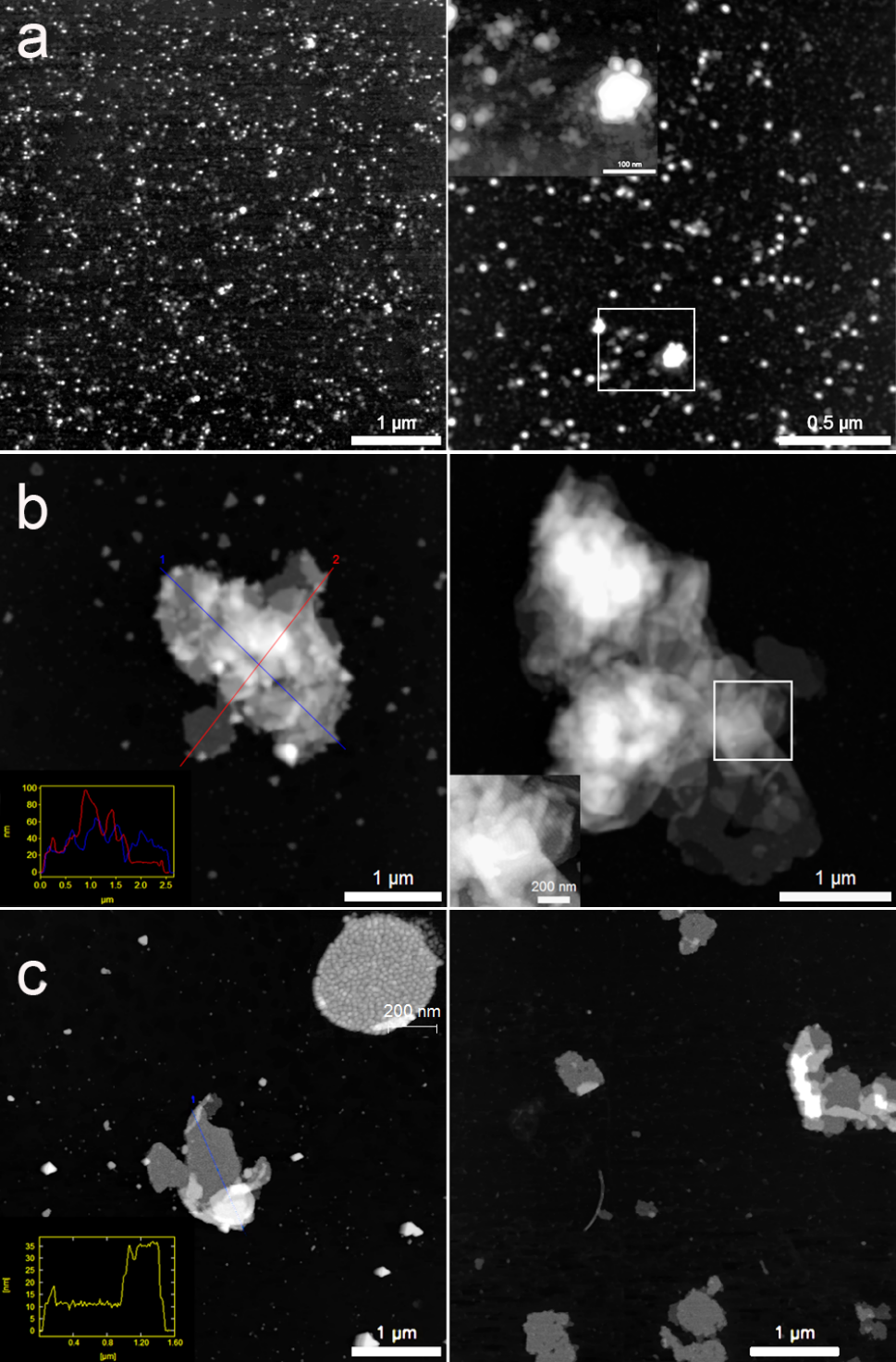


Figure S1. AFM micrographs of grana thylakoid solubilized under different detergent conditions. Ionic concentrations and mechanical treatments remained constant. (a) Triton X-100 solubilization (3.75% for 20 min). Left and right, two different mica locations covered with small grana fragments (≤ 100 nm) suggest over-solubilization. Inset, zoom in on the region marked by the rectangle box. Z scale 0-25 nm. (b) Large and highly variable multilayer membrane patches of thylakoids solubilized with 1.4% n-dodecyl-α maltoside (α-DM) for 5 min (left) or 10 min (right). Scarcity of individual grana discs implies partial solubilization; lack of flat regions prevents further image analysis (right inset). Left inset depicts changes in height across the patch, right inset is a high resolution scan from the region marked by the box. Z scale 0-180 nm. (c) Multilayer thylakoid patches solubilized with 1.4% α-DM for 20 min. Left and right, two micas from two different preparations. Blue line is a cross-section site with a height profile represented in the bottom inset, six different membrane layers form three stacked grana discs can be inferred. Enrichment of individual or low level fused grana discs with distinct edges (top left inset) evenly distributed on the surface was interpreted as a good solubilization condition which preserves membrane structure. Therefore, these conditions were used for further studies. Z scale 0-50 nm.
