# Supplementary material for: Atomic Force Microscopy of Photosystem II and Its Unit Cell Clustering Quantitatively Delineate the Mesoscale Variability in Arabidopsis Thylakoids
Source: PLoS One. 2014 Jul 9;9(7):e101470. doi: 10.1371/journal.pone.0101470 (PMC4090009; doi:10.1371/journal.pone.0101470)
Supplement: Figure S2 — Model selection metrics for the Gaussian mixture model. (DOCX) [file pone.0101470.s002.docx]

Figure S2. Model selection metrics for the Gaussian mixture model. Top: Frequency with which the number of Gaussian components *k* was selected based on Bayesian Information Criterion (BIC), out of 1000 bootstrap replicates; *k*=6 (black bar) was selected with the highest frequency. Bottom: Whisker plots of BIC for Gaussian mixture models fit to 1000 bootstrap replicates; *k*=6 has the lowest median BIC.
