# Supplementary material for: Atomic Force Microscopy of Photosystem II and Its Unit Cell Clustering Quantitatively Delineate the Mesoscale Variability in Arabidopsis Thylakoids
Source: PLoS One. 2014 Jul 9;9(7):e101470. doi: 10.1371/journal.pone.0101470 (PMC4090009; doi:10.1371/journal.pone.0101470)
Supplement: Figure S3 — Histograms of net particle crystallinity by AFM image. (DOCX) [file pone.0101470.s003.docx]

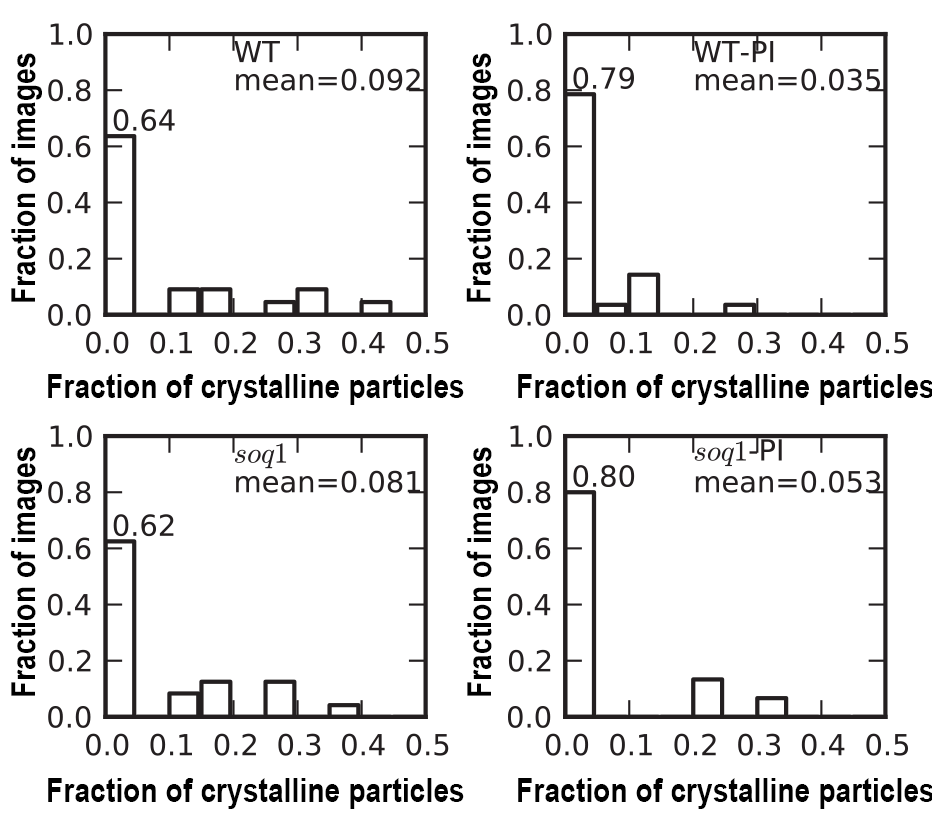


**Figure S3**. Histograms of net crystallinity by particle. Numbers over the left-most bar in each histogram indicate the fraction of images with <5% crystalline particles. The mean fractions are weighted averages, weighted by the total number of particles in each image.
