# Supplementary material for: Atomic Force Microscopy of Photosystem II and Its Unit Cell Clustering Quantitatively Delineate the Mesoscale Variability in Arabidopsis Thylakoids
Source: PLoS One. 2014 Jul 9;9(7):e101470. doi: 10.1371/journal.pone.0101470 (PMC4090009; doi:10.1371/journal.pone.0101470)
Supplement: Figure S4 — Structural models of the six types of crystal arrays clustered by Picolo analysis package. (DOCX) [file pone.0101470.s004.docx]

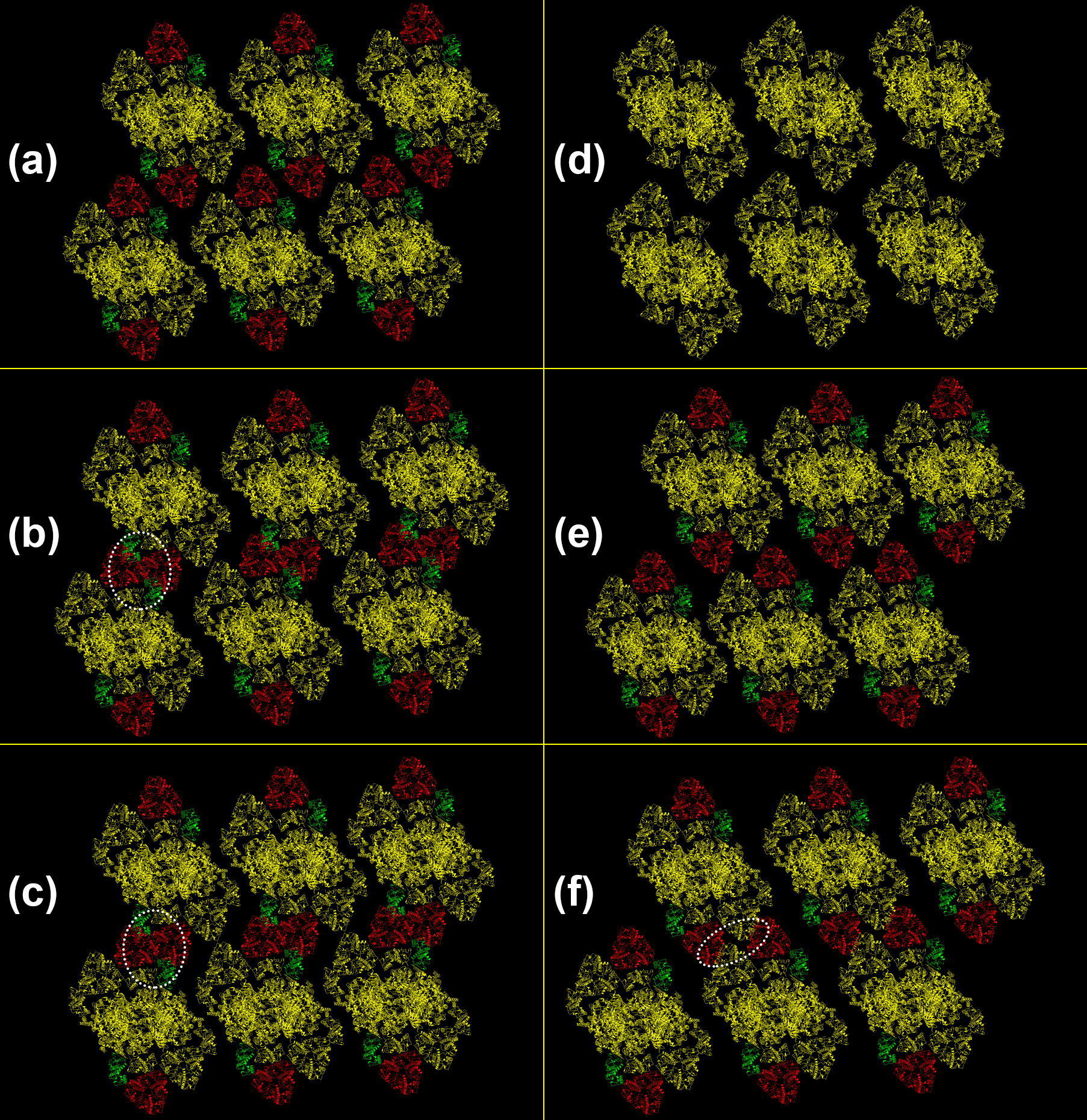


**Figure S4**. Structural models of the six types of crystal arrays clustered by Picolo analysis package. Arrays are displayed as in Figure 2. Supercomplex C_2_S_2_M_2_ was created as described [[1](#_ENREF_1)]. Crystalline arrays were created by fitting C_2_S_2_M_2_ into the average unit cell of each class (Table S1). Reaction center core dimer (C_2_), S-type light harvesting trimers, and minor antenna CP29 and CP26 are represented in yellow; M-type trimers, and CP24 monomers are depicted in red and green respectively. Steric collisions are highlighted by white ellipsoids.

1. Caffarri S, Kouril R, Kereiche S, Boekema EJ, Croce R (2009) Functional architecture of higher plant photosystem II supercomplexes. EMBO J 28: 3052-3063.
