# Supplementary material for: Atomic Force Microscopy of Photosystem II and Its Unit Cell Clustering Quantitatively Delineate the Mesoscale Variability in Arabidopsis Thylakoids
Source: PLoS One. 2014 Jul 9;9(7):e101470. doi: 10.1371/journal.pone.0101470 (PMC4090009; doi:10.1371/journal.pone.0101470)
Supplement: Table S1 — Gaussian mixture model parameters, with bootstrapped 95% confidence intervals. (DOCX) [file pone.0101470.s005.docx]

Table S1

Gaussian mixture model parameters, with bootstrapped 95% confidence intervals.

| Class | Weight | *μ_a_* (CI) (nm) | *μ_b_* (CI) (nm) | *μ_θ_* (CI) (degrees) |
| --- | --- | --- | --- | --- |
| (a) | 0.29 | 20.8 (20.9 ± 0.7) | 25.6 (25.5 ± 0.6) | 71.6 (71.6 ± 1.9) |
| (b) | 0.24 | 21.9 (21.7 ± 0.8) | 23.1 (23.2 ± 0.8) | 74.9 (74.6 ± 3.5) |
| (c) | 0.18 | 20.3 (20.3 ± 1.5) | 24.0 (24.4 ± 2.1) | 76.5 (75.6 ± 11.2) |
| (d) | 0.11 | 18.9 (18.9 ± 0.4) | 23.0 (22.7 ± 1.1) | 83.2 (83.9 ± 2.5) |
| (e) | 0.12 | 19.7 (19.7 ± 0.4) | 28.2 (28.1 ± 0.6) | 68.9 (68.9 ± 2.3) |
| (f) | 0.05 | 22.0 (21.8 ± 0.9) | 26.0 (26.1 ± 1.3) | 63.4 (63.5 ± 8.1) |

| Class | Weight | *σ_a_* (CI) (nm) | *σ_b_* (CI) (nm) | *σ_θ_* (CI) (degrees) |
| --- | --- | --- | --- | --- |
| (a) | 0.29 | 1.27 (0.96 ± 0.49) | 1.01 (1.00 ± 0.37) | 2.50 (2.34 ± 1.25) |
| (b) | 0.24 | 0.74 (0.81 ± 0.35) | 0.78 (0.77 ± 0.25) | 3.03 (2.60 ± 1.27) |
| (c) | 0.18 | 0.60 (0.82 ± 0.45) | 0.93 (1.00 ± 0.49) | 3.21 (2.91 ± 1.60) |
| (d) | 0.11 | 1.01 (0.95 ± 0.33) | 1.74 (1.53 ± 0.52) | 3.85 (3.33 ± 1.72) |
| (e) | 0.12 | 0.69 (0.69 ± 0.21) | 0.41 (0.40 ± 0.35) | 1.86 (1.88 ± 0.67) |
| (f) | 0.05 | 1.18 (1.10 ± 0.54) | 1.53 (1.35 ± 0.54) | 5.86 (4.85 ± 3.09) |
