# Supplementary material for: Atomic Force Microscopy of Photosystem II and Its Unit Cell Clustering Quantitatively Delineate the Mesoscale Variability in Arabidopsis Thylakoids
Source: PLoS One. 2014 Jul 9;9(7):e101470. doi: 10.1371/journal.pone.0101470 (PMC4090009; doi:10.1371/journal.pone.0101470)
Supplement: Table S2 — Comparison of grana complexes height and area parameter distributions from representative patches with different type of packing. (DOCX) [file pone.0101470.s006.docx]

**Table S2.** Comparison of grana complexes height and area parameter distributions from representative patches with different type of packing.

|  | **n** | **Height distribution** | | | | **Area distribution** | | | |
| --- | --- | --- | --- | --- | --- | --- | --- | --- | --- |
|  |  | **A_1_** | **HM_1_±σ_1_** | **A_2_** | **HM_2_ ±σ_2_** | **A_1_** | **AM_1_±σ_1_** | **A_2_** | **AM_2_ ±σ_2_** |
| **Disordered** | 195 | 0.24 | 10.4±0.4 | - | - | 39 | 454±122 | - | - |
| **Crystal a** | 97 | 0.18 | 10.9±0.4 | 0.06 | 12.0±0.4 | 18 | 404±32 | 19.5 | 504±32 |
| **Crystal b** | 190 | 0.09 | 11.5±0.5 | 0.16 | 12.5±0.5 | 24 | 371±59 | 16.5 | 543±59 |
| **Crystal c** | 282 | 0.07 | 10.4±0.4 | 0.16 | 12.1±0.4 | 21 | 331±65 | 18.8 | 478±65 |

n = number of particles, A = Gaussian’s area, M= mean, σ = standard deviation
