# Supplementary material for: Atomic Force Microscopy of Photosystem II and Its Unit Cell Clustering Quantitatively Delineate the Mesoscale Variability in Arabidopsis Thylakoids
Source: PLoS One. 2014 Jul 9;9(7):e101470. doi: 10.1371/journal.pone.0101470 (PMC4090009; doi:10.1371/journal.pone.0101470)
Supplement: Table S3 — Comparison of nearest neighbor distribution's fitting parameters. (DOCX) [file pone.0101470.s007.docx]

**Table S3.** Comparison of nearest neighbor distribution’s fitting parameters

|  | **n** | **A_1_** | **HM_1_±σ_1_** | **A_2_** | **HM_2_ ±σ_2_** | **A_3_** | **M_3_±σ_3_** |
| --- | --- | --- | --- | --- | --- | --- | --- |
| **WT** | 12 | 0.17 | 14.5±1.6 | 0.51 | 18.3±1.6 | 0.28 | 22.5±1.6 |
| **WT-PI** | 18 | 0.20 | 14.8±2.0 | 0.55 | 18.8±2.0 | 0.25 | 22.8±2.0 |
| ***soq*1** | 14 | 0.33 | 14.3±1.6 | 0.43 | 17.7±1.6 | 0.21 | 21.1±1.6 |
| ***soq*1-PI** | 10 | 0.08 | 13.0±2.0 | 0.60 | 17.8±2.0 | 0.31 | 22.1±2.0 |

n = number of micrographs analyzed, A = Gaussian’s area, M= mean, σ = standard deviation
